# Supplementary material for: Microstructural asymmetry in the human cortex
Source: Nat Commun. 2024 Nov 22;15:10124. doi: 10.1038/s41467-024-54243-9 (PMC11584796; doi:10.1038/s41467-024-54243-9)
Supplement: Supplementary file 1 — Supplementary Information [file 41467_2024_54243_MOESM1_ESM.pdf]

Supplementary Materials for
“Microstructural asymmetry in the human cortex”

Bin Wan [1,2,3,4], Amin Saberi [1,4,5], Casey Paquola [4], H. Lina Schaare [1,4], Meike D. Hettwer [1,4,5,6], Jessica Royer [7], Alexandra John [1,4], Lena Dorfschmidt [8,9], Şeyma Bayrak [1,3,4], Richard A.I. Bethlehem [10], Simon B. Eickhoff [4,5], Boris C. Bernhardt [7], Sofie L. Valk [1,4,5]

1. *Otto Hahn Research Group Cognitive Neurogenetics, Max Planck Institute for Human Cognitive and Brain Sciences, Leipzig, Germany.*
2. *International Max Planck Research School on Neuroscience of Communication: Function, Structure, and Plasticity (IMPRS NeuroCom), Leipzig, Germany.*
3. *Department of Cognitive Neurology, University Hospital Leipzig and Faculty of Medicine, University of Leipzig, Leipzig, Germany.*
4. *Institute of Neuroscience and Medicine (INM-7: Brain and Behavior), Research Center Jülich, Jülich, Germany.*
5. *Institute of Systems Neuroscience, Medical Faculty and University Hospital Düsseldorf, Heinrich Heine University Düsseldorf, Düsseldorf, Germany.*
6. *Max Planck School of Cognition, Leipzig, Germany*
7. *McConnell Brain Imaging Centre, Montréal Neurological Institute and Hospital, McGill University, Montréal, QC, Canada.*
8. *Department of Child and Adolescent Psychiatry and Behavioral Science,The Children's Hospital of Philadelphia, Philadelphia, PA, USA.*
9. *Lifespan Brain Institute, The Children's Hospital of Philadelphia and Penn Medicine, Philadelphia, PA, USA.*
10. *Department of Psychology, University of Cambridge, Cambridge, UK.*

Supplementary Materials

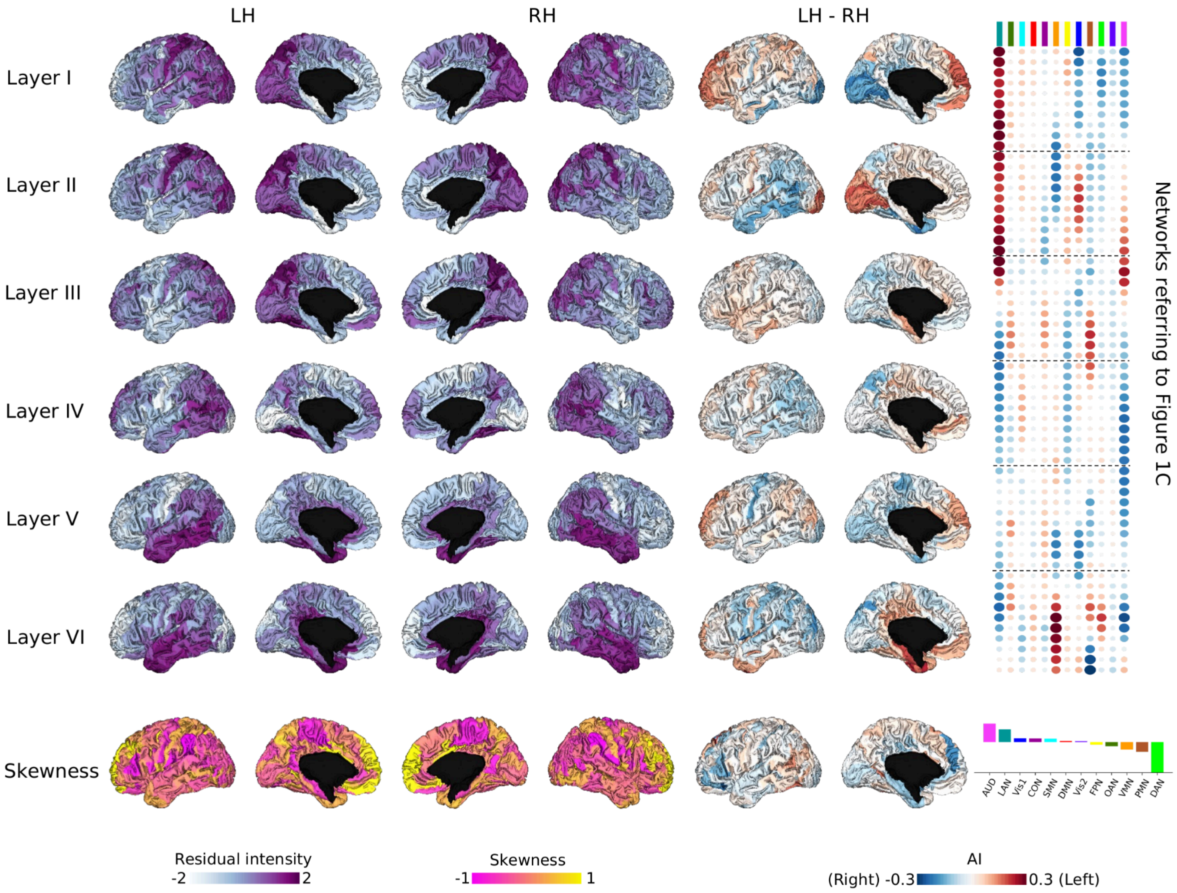

Supplementary Figure S1. Residual intensity values for each layer in BigBrain ($N = 1$).

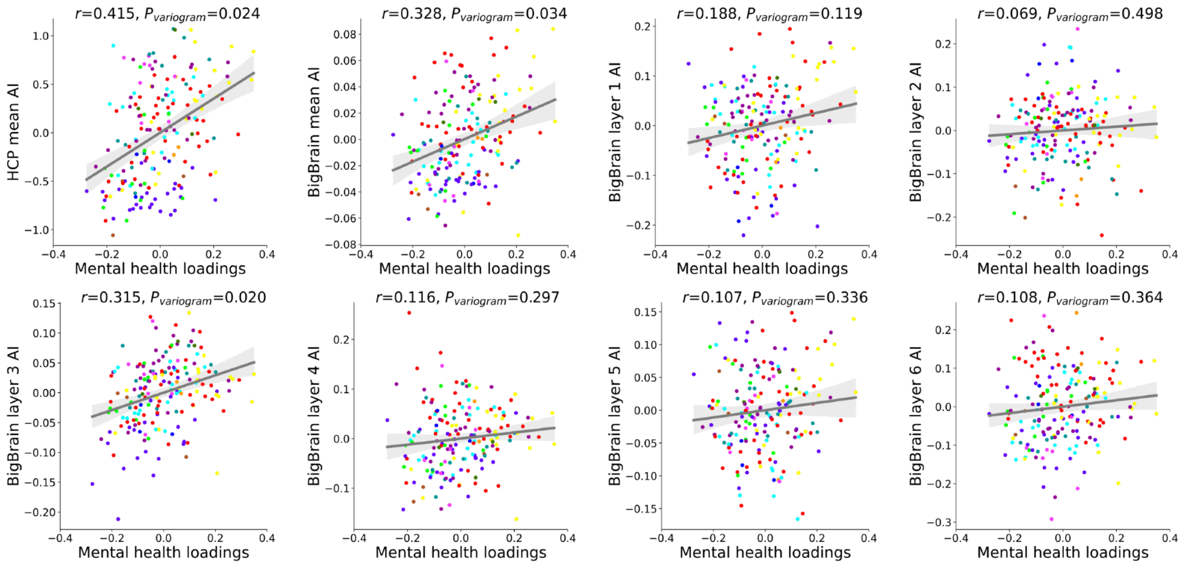

Supplementary Figure S2. Spatial correlation between CCA mental health brain loadings and AI maps (two-sided). Dots are 180 parcels.

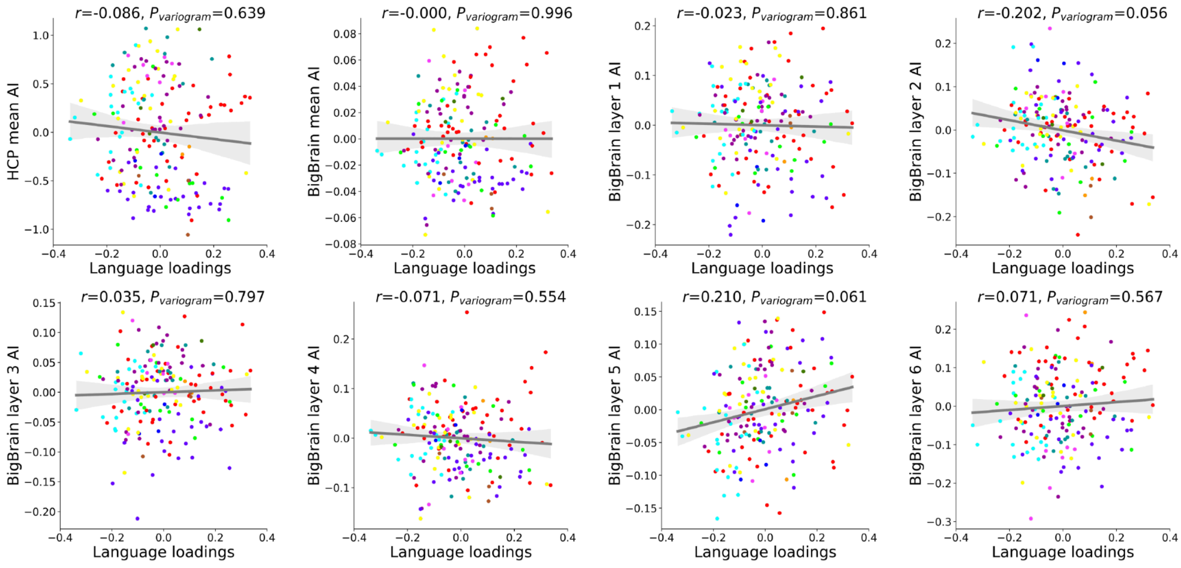

Supplementary Figure S3. Spatial correlation between CCA language brain loadings and AI maps (two-sided). Dots are 180 parcels.

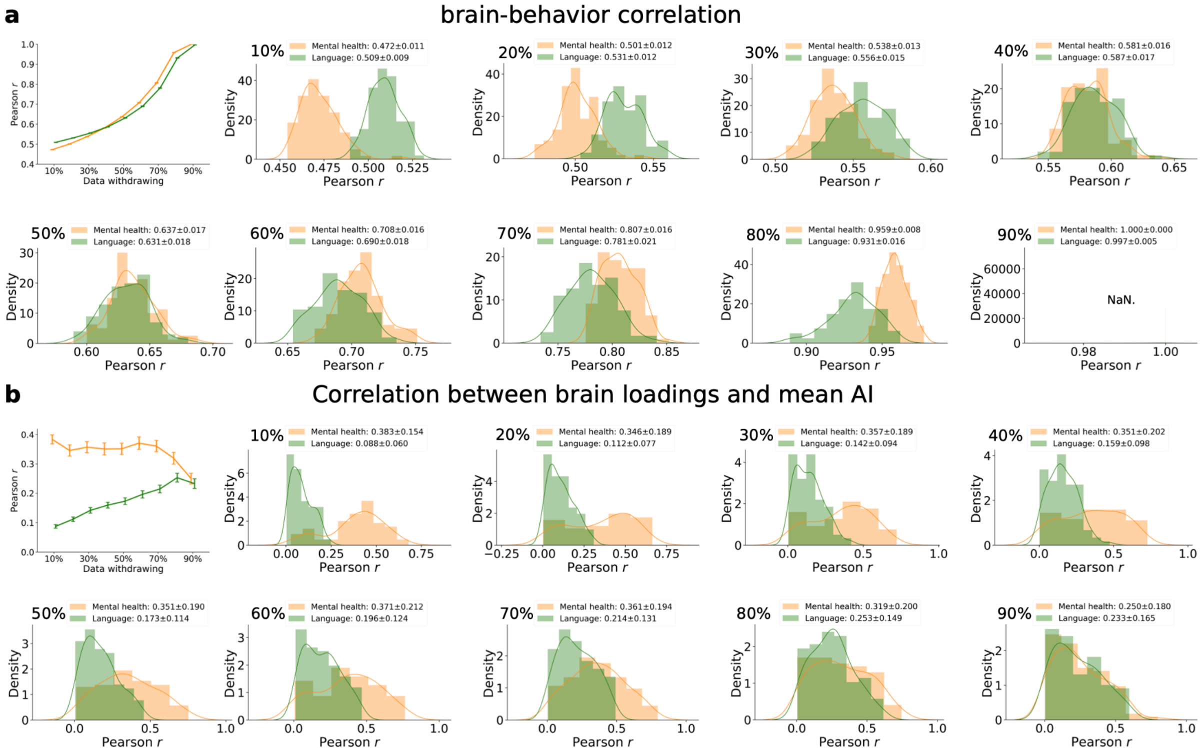

Supplementary Figure S4. Robustness test for CCA using resampling for 1000 times in HCP. **a**. Brain-Behavior correlation of the first latent dimension. **b**. Correlation between brain loadings and mean asymmetry index (AI) of HCP. Percentages are the data exclusion for resampling. Bars are standard errors.

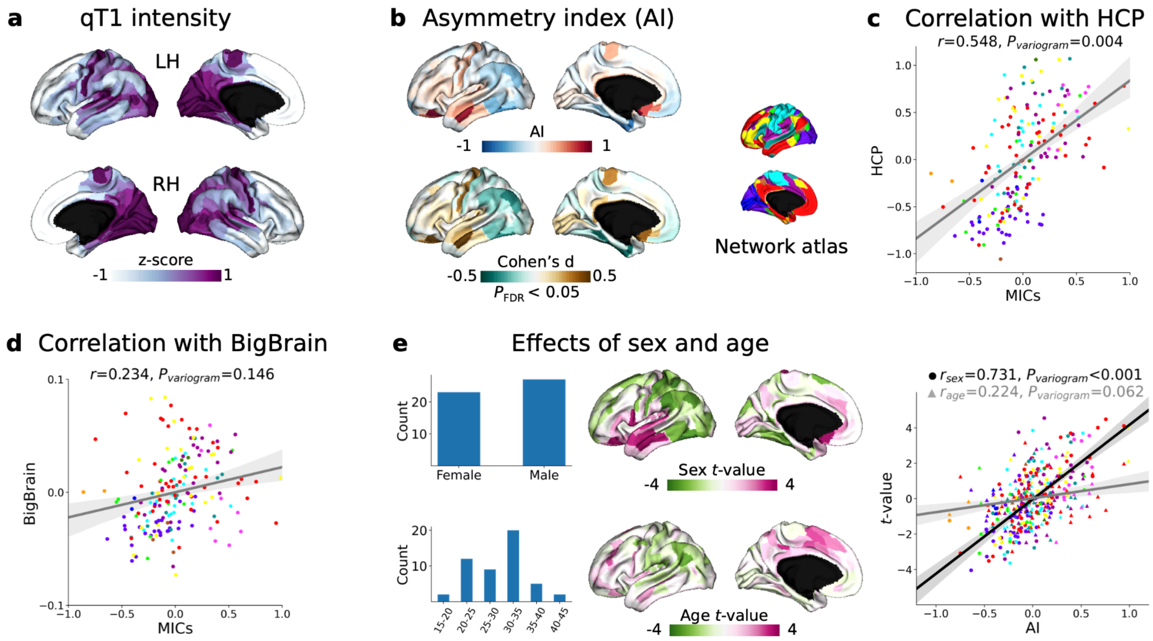

Supplementary Figure S5. Microstructural asymmetry in MICs (N = 50). **a**. Z-score of quantitative T1 (qT1) intensity. **b**. Mean and Cohen's d asymmetry maps of individual intensity. **c**. Mean asymmetry map correlation between MICs qT1 and HCP T1w/T2w. **d**. Mean asymmetry map correlation between MICs qT1 and BigBrain cytoarchitecture. **e**. Effects of sex and age on the asymmetry index and correlation between sex/age effect and mean asymmetry index maps. Dots in the scatter charts are 180 parcels and colored by network atlas.

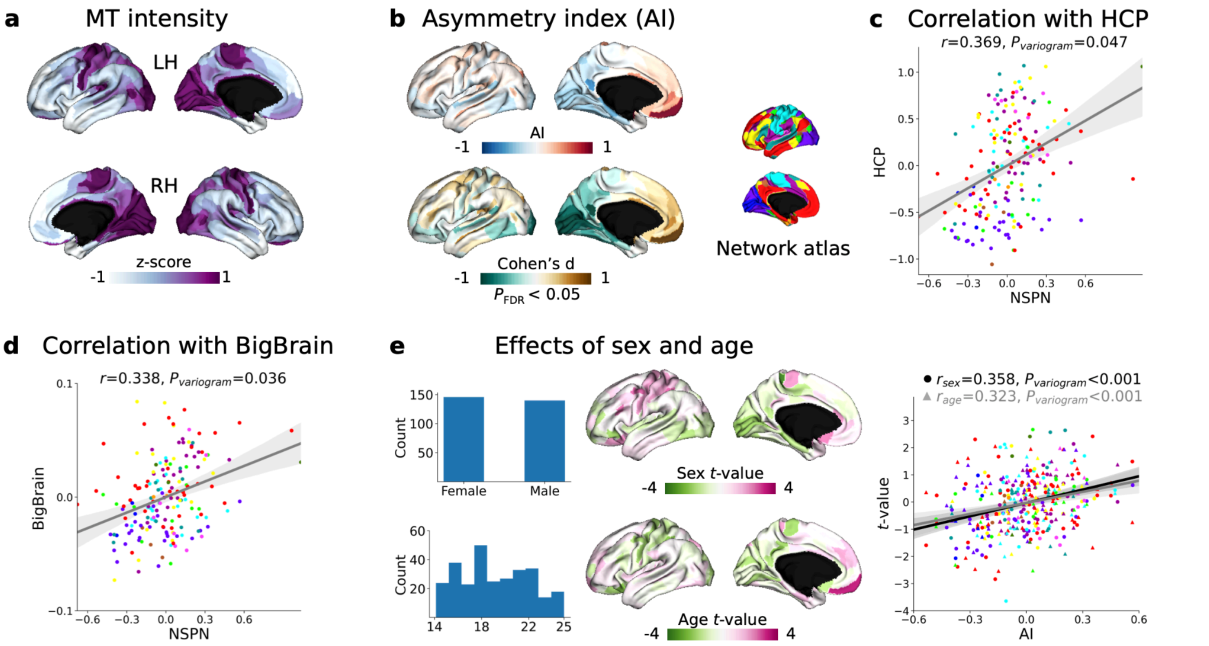

Supplementary Figure S6. Microstructural asymmetry in NSPN (N = 286). **a**. Z-score of magnetization transfer (MT) intensity. **b**. Mean and Cohen's d asymmetry maps of individual intensity. **c**. Mean asymmetry map correlation between NSPN MT and HCP T1w/T2w. **d**. Mean asymmetry map correlation between NSPN MT and BigBrain cytoarchitecture. **e**. Effects of sex and age on the asymmetry index and correlation between sex/age effect and mean asymmetry index maps. Dots in the scatter charts are 180 parcels and colored by network atlas.

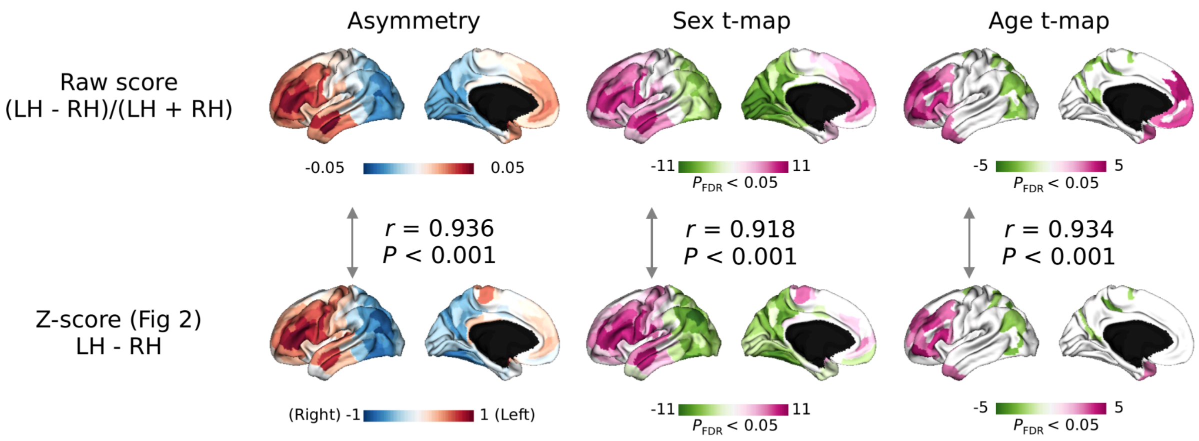

Supplementary Figure S7. Raw score versus z-score for asymmetry index ($N = 1101$).

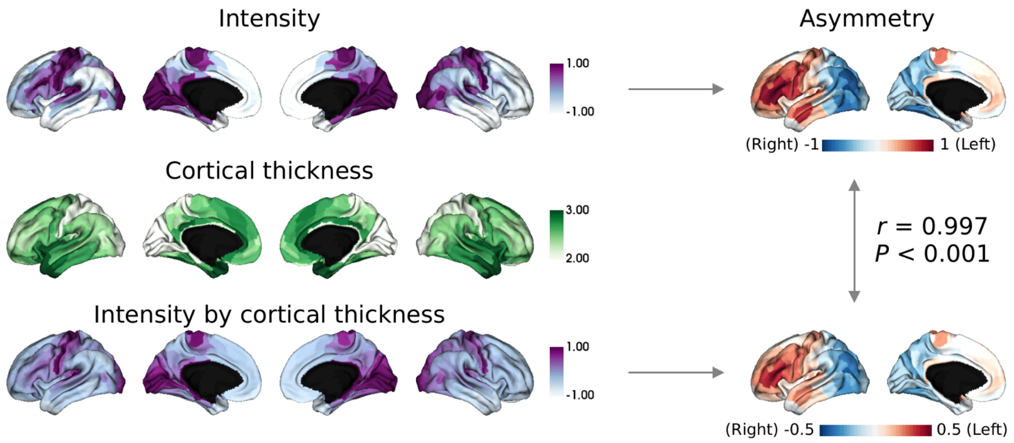

Supplementary Figure S8. Asymmetry of microstructural intensity by cortical thickness (*N* = 1101).

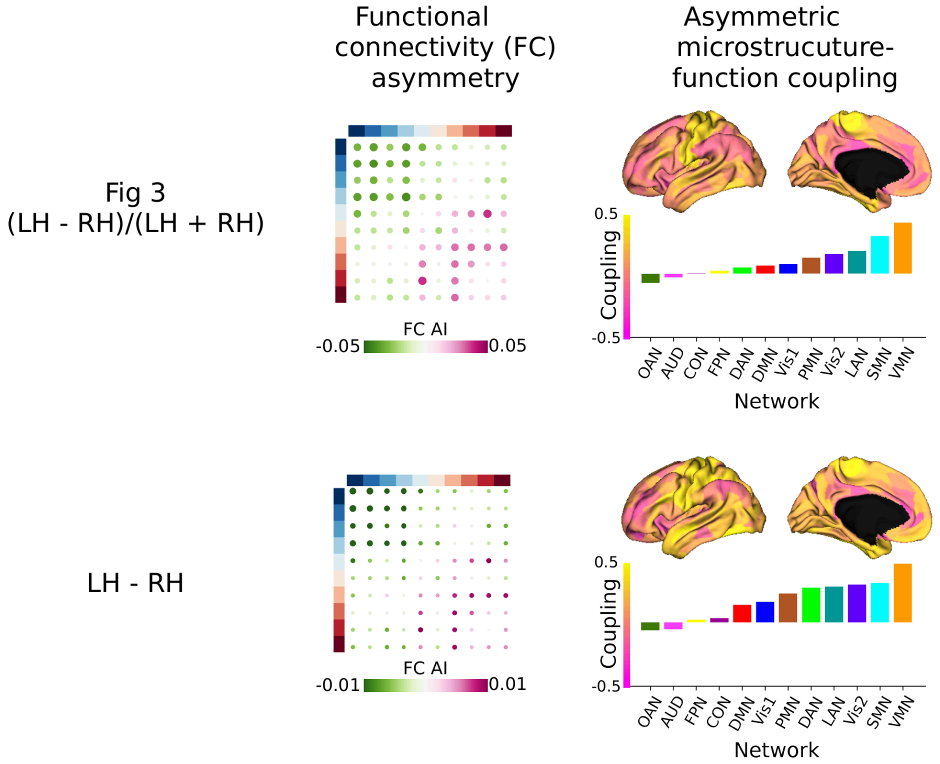

Supplementary Figure S9. Comparisons between original asymmetry (LH-RH)/(LH+RH) calculation and LH-RH for functional data (N = 1004).

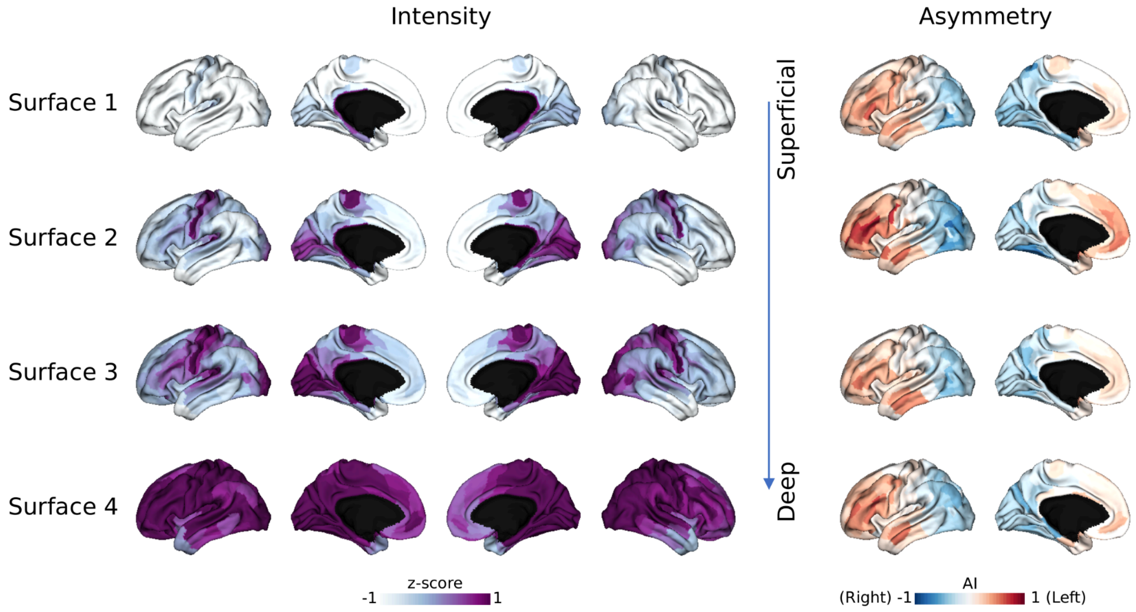

Supplementary Figure S10. T1w/T2w asymmetry across four equivolumetric surfaces (*N* = 1101).

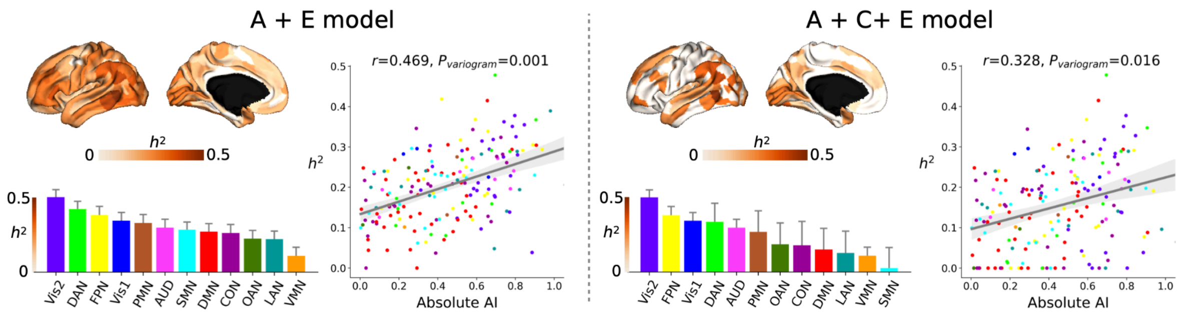

Supplementary Figure S11. T1w/T2w asymmetry heritability in HCP (N = 1101): A + E versus A + C + E model. Dots are 180 parcels.
